# Perceptions of resources available for postgraduate family medicine training at a South African university

**DOI:** 10.4102/phcfm.v14i1.3746

**Published:** 2022-12-20

**Authors:** Neetha J. Erumeda, Louis S. Jenkins, Ann Z. George

**Affiliations:** 1 Department of Family Medicine and Primary Care, Faculty of Health Sciences, University of the Witwatersrand, Johannesburg, South Africa; 2 Gauteng Department of Health, Ekurhuleni District Health Services, Germiston, South Africa; 3 Division of Family Medicine and Primary Care, Faculty of Health Sciences, Stellenbosch University, Cape Town, South Africa; 4 Western Cape Department of Health, George Hospital, George, South Africa; 5 Primary Health Care Directorate, University of Cape Town, Cape Town, South Africa; 6 Centre of Health Science Education, Faculty of Health Sciences, University of the Witwatersrand, Johannesburg, South Africa

**Keywords:** Clinical trainers, decentralised training, family physicians, family medicine, postgraduate training, registrars, resources, faculty support

## Abstract

**Background:**

Clinical training is one of the roles of family physicians (FPs) in decentralised postgraduate training. Effective training requires skilled trainers and sufficient resources. Little is known about the resources available for decentralised clinical training in district health systems in low- to middle-income countries, especially in sub-Saharan Africa.

**Aim:**

To explore FPs’ and registrars’ perceptions of the available resources in a decentralised postgraduate family medicine (FM) training programme.

**Setting:**

Five decentralised training sites affiliated with the University of the Witwatersrand across two provinces in South Africa.

**Methods:**

This qualitative study forms part of a broader project evaluating a FM registrar training programme using the logic model. Semistructured interviews were conducted with a purposive sample of 11 FPs and 11 registrars. The interviews were transcribed verbatim and analysed thematically.

**Results:**

Three themes were identified: ‘Impact of resource constraints’, ‘Family physicians’ skills and knowledge could be further improved’ and ‘Family physicians need additional support to optimise their training role’. The additional resources needed include more FPs, equipment, infrastructure and funding. Knowledge and skills of FPs were reported variable and needed further improvement. Additional support was required from peers, the district management and the university.

**Conclusion:**

Well-resourced decentralised training environments with sufficient skilled trainers and adequate resources are needed to positively influence FP training and supervision, especially in middle-income countries like South Africa.

**Contribution:**

Clinical trainers need adequate resources and support from peers, district management and the university for effective decentralised clinical training.

## Background

The World Health Organization advocates expanding medical education to create socially accountable healthcare workers to meet the needs of communities.^1,2^ Decentralised clinical training (DCT) is a recognised strategy for transforming health professions education to meet community health needs.^3,4,5^ Family medicine (FM) plays a critical role in addressing the health needs of communities through providing primary healthcare (PHC).^3,6,7,8^ Despite many countries having recognised the value of training primary care physicians in decentralised settings,^6,8^ effective training requires sufficient resources and skilled trainers. Decentralised clinical training poses several challenges relating to resource provision and adequate trainer skills, which are accentuated in resource-constrained countries.

The scope and structure of FM and DCT vary widely between high- and low-middle income countries.^3,6,8^ The United Kingdom, United States of America and Canada have established FM training programmes, while India and China are still establishing the discipline because of human resource shortages and a lack of support for PHC.^6,7^ The global situation is mirrored in Africa, where countries like South Africa (SA), Nigeria and Ghana have established training programmes and are collaborating with other countries.^8^ Many African countries, including Rwanda and Tanzania, are still establishing FM training programmes. The focus of FM training in many African countries differs from that in high-income countries because of the high prevalence of a quadruple burden of disease.^9,10,11,12^ Another difference is that FM training in Africa is based in district health systems comprising clinics, hospitals and the community, compared with general practice settings in high-income countries.^3,6,8^ District health–based training requires family physicians (FP) in sub-Saharan Africa to have sufficient skills and competencies to work anywhere in the district health system and function as consultants in district hospitals.^3,7,13^

The upscaling of FM across African countries requires well-equipped district training complexes with adequate infrastructure, information technology, equipment, funded posts for FPs and trainees and enough FPs as clinical trainers.^13^ Other necessary resources include a good patient mix, clinical trainers with adequate skills, knowledge and attitudes and appropriate trainer-to-trainee ratios.^4,14,15^ University mission statements should also align with PHC expectations to allow learning outcomes to be achieved in the district health system outside tertiary institutions.^15^ Decentralised clinical training requires a shared vision integrating a learning culture and clinical training at decentralised sites.^14,16^

Effective clinical trainers and the clinical supervisory relationship between the trainer and trainee are fundamental in FM DCT. The role of the clinical trainer is complex, requiring good clinical reasoning skills and knowledge,^17,18^ sound interpersonal skills and an empathetic and respectful attitude towards patients.^17,19^ The role requires a passion for teaching and commitment to excellence, the ability to establish rapport, communicate effectively with trainees and contribute towards a positive learning environment that reflects trainee needs.^17,18,19,20^ Effective clinical trainers should embody personal characteristics like enthusiasm,^18,21^ honesty, competency and compassion.^20^ They should self-evaluate, reflect on their teaching and adapt the training to the trainee’s learning needs.^19^

The characteristics of individual clinical trainers influence the clinical supervisory relationship between the trainer and trainee, which is regarded as the most vital factor for effective training.^21,22^ This relationship is enhanced by role modelling, trust between the trainer and trainee and the ability to adjust to a particular role.^21^ The potential barriers to a good relationship include trainer–trainee conflicts, power imbalances, hierarchical cultures, a lack of personal contact, limited trainer availability, emotional distancing from trainers, excessive workload, physical environment not conducive for training, a lack of incentives and rewards and insufficient training resources.^21^ Other potential challenges to the supervisory relationship include the multiple roles trainers need to fulfil, possibly during the same clinical encounter, and time constraints because of multiple areas of responsibility.^19^

Decentralised clinical training poses several challenges, even in high-income countries. A study from the United Kingdom drew attention to the impact of heavy workloads and resource shortages on clinical training and highlighted the need for protected time for training and other educational responsibilities.^16^ Trainers and trainees felt unappreciated by the district management and perceived a lack of support from senior clinical managers.^16^ They also felt the district management was unaware of the impact of clinical training in the workplace and was only concerned about meeting clinical targets.^16^

Providing adequate resources for postgraduate training programmes in sub-Saharan Africa is challenging. Previous studies across various specialities, including FM, reported a lack of training standards and locally relevant curricula, unstructured programmes, excessive workloads for trainees, inadequate bedside teaching and reliance on other trainees for learning.^23,24,25^ Another challenge relates to influencing the quality of the training provided by clinical trainers employed by health departments who run the university programmes at decentralised sites.^23^ Other studies described infrastructure and Internet availability shortages in some sub-Saharan decentralised settings,^26,27^ suboptimal conditions of health facilities and educational resources and inadequately maintained equipment.^23,24^ There have been positive reports of trainees in some settings who were satisfied with the infrastructure and equipment availability at PHC clinics in postgraduate programmes^28^ and adequate support from other regional universities, staff commitment and protected time.^24^ South Africa is not exempt from the challenges related to providing sufficient resources and support for DCT. Although no studies on an in-depth evaluation of resource availability could be found, some universities reported having invested in infrastructure and equipment, including information technologies and Internet connectivity.^29^ There is a lack of clarity around the roles and responsibilities of clinical trainers, who provincial health departments and relevant universities jointly appoint.^29^ Previous studies have suggested that DCT in SA could be strengthened by longitudinal relationships between stakeholders, including universities, health departments, health managers and other health professionals.^29,30^

The postgraduate FM training programme at the University of the Witwatersrand in SA has existed for more than 10 years. This study forms part of a broader convergent mixed-methods project evaluating the postgraduate FM training programme using the logic model. The logic model is a causal model based on the reductionist theory that links the inputs, activities or processes, outputs and outcomes.^31,32^ The availability of resources influences the competencies that the registrar achieves during their training. This article reports on the ‘inputs’, namely FPs’ and registrars’ perceptions of the available resources for postgraduate FM training across decentralised sites affiliated with the University of the Witwatersrand.

## Research method and design

### Study design and setting

This qualitative study forms part of a broader instrumental case study evaluating postgraduate FM training programme using open coding. Case studies are useful for exploring phenomena using several data sources and data collection methods.^33^ Instrumental case studies focus on gaining insight into an issue through an in-depth understanding of the case or cases included in the study,^34^ making this approach suitable for researching workplace-based learning and teaching across different contexts.^35^ The setting for the case study is the five decentralised training districts affiliated with the University of the Witwatersrand. Four districts (Ekurhuleni, Johannesburg Metro, Sedibeng and West Rand) are in Gauteng province, while the Dr Kenneth Kaunda district is in the North West province.

Postgraduate FM registrar training takes place in PHC clinics and community health centres in the district. Community health centres are 24 h facilities run by nurses and supported by general practitioners and FPs. Registrars (RGs) rotate for 2–3 months in the district or regional hospital departments. District hospitals are 50–300-bedded hospitals serving a defined population, providing 24 h comprehensive care services, mainly run by general practitioners with some general specialists, including FPs.^36^

The registrars undergo three years of training, including three weekly blocks per year of training at the university and the fourth year of elective rotations. Academic tutorials or skills are taught in groups or individually at regional training centres based in PHCs, community health centres or the district hospital. The trainer roles of FPs at the decentralised sites involve clinical training and supervision.

Clinical training includes discussions of clinical cases and conducting observed consultations and procedures. Academic supervision involves the supervision of assignments, research and various presentations.^37^ The educational supervisory role includes mentoring the registrar, performing various summative and formative assessments and participating in the district training programme, as evidenced in the registrar’s learning portfolio.^37^

### Study population and sampling

The study population consisted of 20 FPs and 21 registrars in the training programme during 2020. There were two levels of purposive sampling. At the first level, FPs and registrars were purposively sampled^38^ because they were best suited to provide in-depth perceptions of the available resources for postgraduate FM training. To ensure broad geographical representation, FPs and registrars from all five training sites were eligible for participation.

The second level of purposive sampling was aimed at FPs functioning in various roles, such as the head of department or unit at FM departments of districts, district education coordinators organising the training programmes and senior and junior FPs. The inclusion criteria for FPs were registration with the Health Professions Council of SA as specialists,^39^ joint appointment by the Department of Health and the university and involvement in registrar training. The FPs recently qualified but not registered as specialists or FPs working as full-time staff at the university were excluded. All second- and third-year registrars were invited to participate in the study. Registrars in the first year of training were excluded as they would not have had adequate experience of the programme. The final sample consisted of 11 FPs and 11 registrars.

### Data collection and analysis

Semistructured interviews were conducted with the 11 FPs and 11 registrars between March and August 2020. The interviews were initially intended to be face-to-face. However, only eight were conducted in-person because of the coronavirus disease 2019 (COVID-19) lockdown. The remaining 14 interviews were conducted virtually using Zoom (Zoom Video Communications, Inc., San Jose, California, United States of America) or Microsoft Teams (Microsoft Corporation, Redmond, Washington, United States of America). The researcher made field notes and used an interview guide (see Appendix 1). Each interview lasted from 80 min to 90 min. All interviews were audio-recorded and transcribed verbatim, and the transcripts were checked for fidelity.

The transcripts were analysed using the Braun and Clarke six-step approach of data familiarisation, coding, searching for the themes, naming and reviewing the themes and writing the report.^40^ MAXQDA 2020 (Verbi Software, Berlin, Germany) was used to manage the analysis. The interviews with each group of participants were conducted while coding the transcripts and comparing and contrasting the codes and themes, as described by Tesch,^41^ until no new codes and themes were identified, suggesting that data saturation^40^ had been reached. Although no new themes were identified after nine interviews in each group of 20 and 21 participants, two more participants from each group were interviewed to promote data saturation. Extracts from participants’ comments are identified using RG for registrar and FP for family physicians. The results of this study are presented in a thematic map. A thematic map is helpful for identifying themes, subthemes and interconnections between themes and subthemes.^40^

Several methods were used to improve the trustworthiness of the findings. The coding scheme was developed over several iterations of checking and rechecking by all authors, with their different areas of expertise, discussing the coding system until agreement was reached, improving intercoder reliability.^42,43^ Reflexivity refers to how the researcher’s role, their personal background and experiences potentially influence their interpretations of the data, thus shaping the direction of the study.^42^ The first author was previously an academic coordinator at one of the decentralised sites, which means that she had first-hand knowledge of the registrar training programme. As a colleague of the FPs and supervisor of some registrars who participated in the study, the first author continually reflected on issues like collegiality and power dynamics to avoid influencing the interviewees and the coding process. The first author’s reflexivity was also augmented by keeping a record of the coding process and interpretations in MAXQDA for discussion with her co-authors. Such reflexivity raised her awareness and allowed her to mitigate potential biases in the study. Theme development and naming were cross-checked by the co-authors, who are experienced qualitative researchers, to enhance the credibility of the findings. Purposive sampling^38^ of each group of participants and triangulating^44^ the findings from the two groups, that is FPs and registrars, further improved the credibility of the findings. Detailed descriptions of the study methods enhanced the transferability of the study and keeping an audit trail improved the dependability, thus improving the trustworthiness of the study findings.

### Ethical considerations

Ethical approval was obtained from the Human Research Ethics Committee (HREC Medical) of the University of the Witwatersrand (ref. no. M191140). Permission to conduct the study was obtained from the Gauteng and North West province district research committees. Informed written consent was obtained from study participants. The research was carried out following the Helsinki Declaration.

## Results

The participants’ characteristics are shown in Table 1. The FPs ranged in age from 41 to 60 years and had more than 5 years of registrar-training experience. Many FPs had attended short training courses, but none had any formal medical education training. The registrars’ ages ranged from 30 to 60 years. Six were in their third year of study and five in the second year. All the registrars had some PHC experience before joining the programme.

**TABLE 1:** Training and experience of family physicians and registrars.

Family physicians	*n*	Registrars	*n*
**Age**		**Age**	
-	-	≤ 30	3
-	-	31–40	6
41–50	8	41–50	1
51–60	3	51–60	1
**Years of training experience**		**Year of training**	
1–5 years	3	Year 3	6
> 5 years	8	Year 2	5
**Current position in the district**		**Primary healthcare experience before joining the training**	
Family Physician – Grade 1	2	Community service doctor	4
Family Physician – Grade 2 (5 years’ or more experience)	6	Worked for 3–5 years in clinics as a medical officer	3
Family Physician – Grade 3 (10 years’ or more experience)	3	Worked less than 1 year in the clinics as a medical officer	2
Head of Unit or Acting Head of Unit of the district	4	Worked in an HIV clinic	1
Current district education coordinator	3	Worked in private practice	1
Previous district education coordinator	3	Primary healthcare patients seen at the district or regional hospital	1
**Postgraduate qualifications other than Family Medicine (degree or diploma)**		**Postgraduate qualifications other than Family Medicine before joining the programme (degree or diploma)**	
Yes	8	Yes	3
No	3	No	8
**Postgraduate degree or diploma in medical education**			
No	11	-	-
Yes	0	-	-
**Medical education training courses attended**			
Yes	9	-	-
No	2	-	-

HIV, human immunodeficiency virus.

**FIGURE 1:**
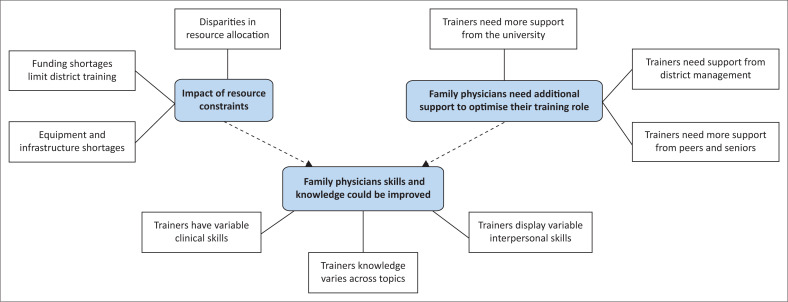
Thematic map.

The three themes and subthemes identified are represented in a thematic map (see Figure 1).

### Impact of resource constraints

Most participants reported shortages of training tools, infrastructure, Internet availability and funding. Both groups of participants expressed the need for improved university support in recruiting more FPs to district training sites. They felt the university could improve support to address these shortages.

#### Equipment and infrastructure shortages

Both registrars and FPs commented on equipment challenges, including the shortage of mannikins. Some essential equipment were unavailable or nonfunctional. These prevented registrars from practising skills they learnt at the workplace:

‘You don’t have peak flows, you don’t have ECGs [*electrocardiograms*], like those basic things that should be in a clinic you don’t have, and then that makes it difficult in terms of your doing things on a daily practice. You know, like even your ophthalmoscope and otoscope, it’s not like working, so you have to like [*to*] carry all your own equipment just to like examine a patient properly.’ (RG 10)

Family physicians expressed their concerns about not receiving adequate equipment from the university while training in districts:

‘In terms of learning materials, for example, whenever we need ECG, they don’t give us that one, or sonar machine, or something that is helpful, or prepare anything of [*sic*] skill improvement or opportunities for [*the*] family physician. So, we do it voluntarily, even though there is not enough support.’ (FP 2)

Both FPs and registrars identified several challenges regarding the limited training spaces, consultation rooms, projectors or laptop availability:

‘It would be nice just to have some room that was dedicated to us from a certain hour … from hour to hour, it would be great. Because that’s why we end up coming home on an academic day because I don’t want to sit in a room where there’s no desk and there’s no … so DH [*district hospital*] 5 in the family medicine room there is no Wi-Fi … there’s just no signal, so you are like in a cave.’ (RG 11)

Family physicians and registrars expected more faculty support to optimise infrastructure at training sites and for joint appointees to access the tools needed for training:

‘The university does not own any infrastructure here. And we will be glad if they can help [*with*] the infrastructure here.’ (FP6)

‘Even in terms of finance, in terms of competence, in terms of cell phone, in terms of any learning, we don’t have support from the university.’ (FP 2)

Variable Internet connectivity was a major challenge. Registrars regularly used the Internet in most districts, but in some districts, they were frustrated by the lack of consistent and stable online access:

‘It’s actually very interesting that you mention the Wi-Fi things because nowadays there’s a lot to do online, the EML [*Essential Medicine List*] is available online and many other apps that one can use for their work. So ja, that’s maybe something to start with, to have Wi-Fi available for practitioners to be able to use those tools.’ (RG 6)

Most registrars and FPs felt that the university needed to address Internet availability at training sites:

‘I think those like your internet, maybe the Wits University could organise that but at the district level, and that would be of help.’ (RG4)

Some FPs mentioned a lack of communication from the university before the Internet installation in some districts, which limited its utilisation. According to them, the university should have consulted with district management before installing Internet connections to maximise its usefulness to registrars and medical students rotating at these sites:

‘[…*T*]hey put it [*Wi-Fi*] only in the district centre there, on the fourth floor. But where the students are. It’s not … I mean, you have students coming here, but there’s no Wi-Fi.’ (FP1)

In contrast, FPs and registrars in a few districts felt that they had adequate equipment and infrastructure. Both groups of participants in those districts appreciated the availability of a well-equipped regional training centre with tools to support training activities:

‘Yes, infrastructure – we have a nice regional training centre, and … it’s opened a library now and there’s access to computers, so, I think for the training site, yes, there’s good infrastructure.’ (RG8)

‘Where we are, we’ve got instruments and things to help them to enhance learning. There are mannequins there, and then a lot of other things that are … we’ve got a library there where they can get books for family medicine, projectors, and all that.’ (FP 8)

#### Funding shortages limit district training

Family physicians referred to insufficient funding support from the university and the district management, which meant that the Department of Health was responsible for most funding. Some FPs mentioned the inadequate clinical trainers’ salaries or travel reimbursements but acknowledged that the university provided doctor’s bags stocked with the necessary equipment at the training sites. Registrars pointed out that the district management provided limited funding for continuous medical education activities or workshops:

‘You see, that’s the problem, because the university does not pay anything. That’s my understanding. And I think that is where the problem is with the [*provincial*] department. Because the Department [*of Health*] is paying everything, and I think Wits doesn’t pay anything.’ (FP 11)

Shortages of FP posts at many training sites negatively impacted the training. Family physicians expected the university to recruit more FPs to address this. Other FPs felt more full-time staff were needed for effective DCT:

‘You do not supervise as should be the ideal situation because we don’t have enough doctors, or family physicians to work alongside a registrar in the clinic.’ (FP5)

‘I was able to build it (FPs) up to five … but now you can’t get additional posts.’ (FP9)

‘Permanent professors, we need people appointed full-time to make a difference.’ (FP6)

Registrars indicated a lack of awareness of how the university or district management funds training in the districts:

‘I’m not really sure about how the university funds the district regarding things.’ (RG10)

In contrast, most registrars valued the university bursaries towards their tuition fees:

‘And when it comes to the assistance we get with the bursaries, that’s tremendous, that’s big, that’s a great assistance we get with the bursaries [*sic*].’ (RG6)

#### Disparities in resource allocation

Many registrars felt that training opportunities differed across districts because of unequal resource allocations. According to them, the university may not be aware of differences in the resources available across sites:

‘I doubt the university know[*s*] that what you learn in DT [*district*] 1 is totally different from what people are learning in DT3. So, I don’t think they are involved, [*that*] they actually go and check what is it that you really do in DT1, what is it that you really do in DT 3, and what works in each and every district.’ (RG2)

They felt that the university needed to advocate the standardisation of resources and training across the districts:

‘So, you’ll get more training depending on which district you are in … or you’ll have more resources depending on which district you are in. For example, in DT1 we have the luxury of having many family physicians, whereas in another district they have two or three. So, if you’re in that district, you are limited in terms of the amount of supervision and training you can get as opposed to another district which has more people available for supervision and training. And resources as well. Maybe some districts may have better resources, better equipment than others. So that should be standardised by the university, in my opinion, so that in [*sic*] every site, the registrars get the best out of the training.’ (RG1)

### Family physicians’ skills and knowledge could be improved

Most trainers displayed good clinical knowledge and skills but required further training to improve the necessary skills for their training roles. According to most registrars, although FPs demonstrated good attitudes while engaging with registrars, their interpersonal skills could be improved.

#### Trainers’ knowledge varies across topics

Most registrars felt that FPs had adequate knowledge, although this was dependent on previous clinical experience. Some registrars received more relevant information from junior FPs, because of their recent experience in registrar training, than from older FPs. The FPs also agreed that previous experience enhanced their knowledge, and training registrars forced them to keep their knowledge current:

‘It has actually helped me to go back reading a lot, looking at [*the*] evidence, looking at the books, trying to get more information and credible and evidence-based information so that I would be able to teach the right thing.’ (FP 3)

#### Trainers have variable clinical skills

Family physicians and registrars were of the view that FPs’ clinical skills expertise varied. Some FPs displayed robust research and clinical-governance skills, whereas others were more skilled in clinical training. Most participants felt that the FPs usually demonstrated excellent skills in integrating FM principles and tools during training:

‘I think it’s a strength that I’m able to take them through how different resources, managing resources in a very constrained environment, as well as using all the family medicine tools, family-oriented care, doing family conferencing. So those are my strengths, practising the family practice tools have really been a good strength.’ (FP10)

Most FPs felt there was room for improving their clinical skills and struggled to train registrars in skills that they were not regularly practising themselves:

‘If you do not have your daily practice, then it’s very difficult to keep your hand in and obviously more difficult to train.’ (FP5)

#### Trainers display variable interpersonal skills

Both FPs and registrars said that FPs tried to maintain good interpersonal skills by engaging registrars, sometimes extending their concern to personal and family issues that could impact registrars’ training:

‘My strength is that … you see, from a distance, I’m able to pick up the difficulties that the registrars are having very quickly, which is something that I think I’ve kind of, like, grown over time.’ (FP11)

‘Sometimes [*we*] discuss any issues that I have, including academic, including personal. And if I have concerns or I have things that need to be sorted she would guide me where she can.’ (RG9)

In contrast, some registrars felt that, in some instances, stronger interpersonal relationships were needed:

‘Whereas with senior doctors, it’s more about just work, work, work, work, work, work, work all the time, and you sort of don’t relate because you’re in different fields of life.’ (RG1)

### Family physicians need additional support to optimise their training role

While some FPs perceived a lack of support for their clinical trainer roles from the district management and university, others reported a lack of support from their peers and seniors. The FPs highlighted the advocacy role of the university to obtain support from the Department of Health to create FP posts and provide adequate training. Participants also expressed the view that the university should be more involved in district training by making more regular site support visits.

#### Trainers need more support from peers and seniors

Family physicians perceived that there was a lack of support from their peers or seniors for training in some districts. They wanted more peer mentoring, especially between junior and senior trainers:

‘I think we’re all just so busy and trying to keep our heads above water, that we don’t always support the people around us. And I mean, it is a department where we’ve got young people coming through now and I think older people that aren’t going to be around for too much longer and they’ve got wonderful skills to impart, so I think clearer mentorship would be better.’ (FP 7)

Some FPs felt that more equal workload distribution would reduce the possibility of FPs in some districts being overwhelmed by training:

‘I often get lumped with kind of having to co-ordinate the whole programme and deliver it, which becomes quite heavy, and you also want other people’s input and other people’s brains.’ (FP7)

There was also the view that senior supervisors did not provide the necessary support for training:

‘You know, locally, the senior supervisor has a lot of issues using time for academic [*registrar training*]. You mustn’t put it in your work schedule, so then now you end up using your private time to do academic activities, in terms of outside of the work.’ (FP1)

Although most participants felt that FPs exhibited commendable passion and diligence in their teaching, many registrars were concerned about FPs not being accessible when needed because of the multiple roles and responsibilities they were managing simultaneously:

‘As we do not see them a lot. I do think that one of the areas in our district that can do with some improvement is the fact that we do not see our family physicians as often as we would like to see them.’ (RG8)

#### Trainers need more support from district management

Most participants were concerned that the district management did not fully understand and acknowledge the existence or value of the FM training programme, which negatively impacted the training:

‘First of all, let’s start from the recognition of the training programme. There needs to be clear recognition of the training programme. But that one is something which is … you are not even sure whether it’s been recognised as something … as part of what is going on in the district.’ (FP 11)

The lack of recognition of the programme could have contributed to registrars’ perception of a lack of support or encouragement in performing FP roles, which negatively impacted registrar training:

‘I don’t think there’s a real understanding of the point of family medicine and the point of family physicians and the role that we have to offer, in terms of mentorship and teaching students and really creating facilities to be academically inclined. I think from that point of view it’s quite disheartening because I don’t think they really see the role of family physicians.’ (RG 10)

#### Trainers need more support from the university

Most FPs had strong views on the university’s advocacy role of engaging with the provincial Department of Health to clarify and recognise the role of FM in the district:

‘The only thing I would want the university to do more is more of getting directly involved with regards to negotiating with the district, with the Department of Health, for them to … because there is no way, there’s no way most of these challenges with regards to synergy between the university and … within us and the district will be solved if the university doesn’t come in the forefront. Because they should be the one that will go and explain to them that this is what we are all about; this is why we have this training complex here; this is why we have this training platform here, and all that, so they will understand better.’ (FP 3)

Most FPs raised concerns about requiring more support for training and for their growth and development as trainers from the university. They felt isolated in the districts, with minimal involvement from the university:

‘Well, I think the university, personally, for me, is they’re just there to keep on passing the buck. People must just do the work. They don’t … [*sighs*] the level of interest in terms of knowing, number one, if you are able to do the work, is not there.’ (FP 1)

One registrar echoed the sentiment about the lack of university involvement:

‘The university is not really involved in the district training programme, so there’s not much support from the university in the district.’ (RG1)

Many FPs requested more mentoring from faculty to develop as trainers:

‘But I think, because we’re affiliated to the … we’ve got joint appointment[*s*] with the university, I think we should have more structures, mentoring and growing [*sic*] for supervisors.’ (FP6)

While some FPs acknowledged that existing courses offered by the university or other bodies supported their professional development, others wanted more courses to function optimally in their training and supervisory roles:

‘I would love to be trained in other things, perhaps assessing exams, and go for some other training to improve myself to be able to be a better medical trainer.’ (FP10)

Family physicians also expressed a need for the university to conduct faculty support visits at the sites to assess whether the training standard is appropriate:

‘So, I think the university’s part needs to be just that they need to come out and check and see whether the facilities actually meet the standard of training that the university expects.’ (FP 11)

## Discussion

This study explored South African FPs’ and registrars’ perceptions of the resources available in a decentralised postgraduate FM training programme. The findings contribute to the extant literature on the appropriate training of doctors to equip them to meet communities’ healthcare needs. Most of the FPs who took part had several years of experience and had occupied various roles in the programme, contributing to the robustness of the findings:

Adequate resources are fundamental to improving postgraduate training. Most participants had experienced severe resource shortages across all five training districts, relating to equipment, infrastructure, funding and Internet connectivity. A well-equipped training complex is a fundamental requirement to promote successful learning.^13,14,15^ Although previous studies have reported inadequate material resources at DCT sites in sub-Saharan Africa,^23,24,25,29^ they did not explore individual resources such as equipment, Internet access, infrastructure and funding options available for training and trainer support in postgraduate training, as this study did.

The lack of equipment, mannikins and Internet access hindered the registrars from practising their learnt skills. For example, comprehensive chronic care of a hypertensive and diabetic patient includes a complete eye and foot exam and an ECG as part of an annual check-up. The lack or nonfunctionality of basic equipment like ophthalmoscopes, ECG machines or monofilaments hindered training in most districts. Although the primary responsibility for equipment availability lies with facility managers who are part of the district management, the university needs to ensure the training standards at these facilities. The availability of adequate equipment impacts the standards of care provided to patients attended by registrars during training. Registrars’ ability to access the latest management guidelines for any clinical condition, which forms part of their learning, requires Internet access at their workplace, which was constrained at these sites. Previous studies reported that registrars mainly depended on their clinical knowledge at PHC facilities because of the unavailability of point-of-care tests.^25^ The availability of point-of-care tests depends on ideal clinic standards^45^ but is still inadequate to achieve adequate skills and competencies for registrars at PHC facilities. The provincial departments of health, Wits University and district management could play an advocacy role to address these resource shortages to improve registrar training, thus improving the standard of PHC services they provide in the communities. There should be minimum prescribed standards, including resources for accreditation of the district training sites by the Health Professions Council of SA in postgraduate DCT, as required for undergraduate training. Districts and universities will then be compelled to equip training sites with the resources needed to maintain training standards.

While adequate resources are fundamental, skilled trainers are vital for effective postgraduate training. The FPs displayed variable personal attributes and physician, teacher and personal characteristics, in contrast to what is ideal as described in previous studies,^17,18,20^ suggesting a need for further improvement. The trainers demonstrated varying levels of clinical knowledge and clinical-reasoning skills across topics. Most FPs could engage and mentor registrars during their trainer–trainee relationships, even on personal issues when necessary, an essential attribute for clinical training.^21,22^ Better communication skills could enhance the trainer–trainee relationship, especially with senior trainers, as mentioned by some registrars. The issue of FPs’ lack of availability and accessibility was regarded as an example of ineffective supervisory behaviour,^46^ which could be attributed to their multiple roles and responsibilities, as seen in other studies.^37,47^

Many FPs struggled to train registrars on clinical skills that they did not practise regularly, indicating a need for ongoing training opportunities to maintain and update their skills. While some FPs were based at community healthcare clinics or PHC clinics, others were based at the district hospital, which could explain the differences in their skill levels. Family physicians also said they needed the university to provide and maintain a supportive environment to update their training and supervisory skills. However, FPs agreed that attending training courses organised by the SA Academy of FPs helped them grow better as trainers. More training opportunities to strengthen the trainer role can be made available as workshops, short courses or tool kits, as in high-income countries.^16,48,49,50^ Different teaching models^19^ developed in high-income countries have been adapted to train FPs in decentralised clinical settings. Most trainers had attended courses conducted by the SA Academy of FPs or the university, but more consistent training is preferable to once-off training sessions. Peer mentoring or co-teaching on teaching, assessment, feedback, interpersonal skills and supervisory behaviours as critical skills for effective training and supervision^46^ by more experienced trainers could be another strategy. These skills were perceived as insufficient among FP trainers in this study.

The lack of teamwork, task sharing and understanding of each other’s roles were concerns expressed by both FPs and registrars. The need for robust support among peers, seniors and supervisors is vital for sustainable DCT. While other studies described successful teamwork in a few SA training districts,^37,51^ the teamwork at the study sites needs to be strengthened. The exchanging and sharing of knowledge and skills between peers, especially when some have good supervisory skills in research and clinical governance and others are more skilled in training on common clinical conditions, could enhance training. This shift in the clinical trainers’ mindset in taking responsibility for training with innovative ways of collaboration will significantly strengthen the DCT sites.

Even where adequate resources and skilled trainers are present, support from high-level stakeholders is essential for the successful implementation of training programmes. Most participants were concerned about the lack of support from provincial health and district management departments for FM training. Perceived poor support negatively impacted FP and registrars’ training roles and responsibilities. The findings were similar to previous studies done in high-income countries^6,16^ and sub-Saharan Africa.^3,8,52^ Postgraduate FM training is well established in many SA universities, leading to other African countries developing their training programmes. The poor health system support has been highlighted previously,^8,29^ but the situation remains largely unchanged, as this study also suggests. As more doctors are joining this discipline, greater attention is required to strengthen support from various key stakeholders. Family physicians, when qualified, are based in district health systems to work, train and lead the PHC team, supporting primary-care nurse practitioners, general practitioners and other health-worker cadres, improving the quality of patient care.^53,54^

The multiple responsibilities of FPs jointly appointed by the district and university may negatively impact the effectiveness of clinical trainer role, as described elsewhere.^29^ Better clarification of the role of the trainer by the district and university will help meet expectations on training standards, as found in other SA universities.^25,30^ Building relationships between relevant stakeholders (such as national and provincial health departments, the university and district management) was vital, according to most participants. The overall advocacy role of the university to lead DCT by regular engagement between all stakeholders was highlighted. Enacting these advocacy roles by the university could help clear ambivalence regarding FP training and roles that still existed in academic and political circles.^52,53^

Regular FP engagement by the university or the Academy of FPs and capacitating them for training and supervision, rather than once-off attending training courses, will act as enablers in trainer roles. A ‘see one, do one and teach one’^55^ approach may not work in training and supervisory roles. Although it is expected at times, trainers need constant mentoring and support to perform their training roles effectively. Formative assessment site visits by the SA Academy of FPs as a follow-up after clinical trainers have attended courses could augment clinical trainers’ confidence. Previous studies also suggested the need for ongoing support for clinical trainers involved in DCT.^56^ Regular support visits to these decentralised sites by the university were vital, as suggested by participants. This could assist in achieving expected training standards and acquiring resources.^57^ Family physicians and registrars suggested recruiting more full-time trainers by the university to strengthen DCT. The FP trainers, if newly recruited, should be based anywhere in the district health system, either in PHC or district hospitals, rather than at the regional or tertiary hospitals or the university, acquiring more hands-on patient care experience, capacitating them to develop as clinical trainers.

Another strategy for better university support for DCT could be regular engagement between the university and the FM division, on the joint staff roles, and sharing small successes achieved in DCT, which could improve understanding of FM as a discipline and the FP roles at the university level. Such understanding may result in additional university support, consequently prioritising more funding and resource provision. Available district funds could be used efficiently with added university support to develop successful training sites. For example, the availability of a sonar machine at a community healthcare facility augments the training and transformative learning experience of registrars while they perform an ultrasound on real patients. Registrars can achieve superior competency levels by repeatedly practising these core skills under FP supervision. Registrars can also teach this skill to medical students rotating in PHC contexts, allowing them to practise teaching skills in an authentic context.

Family physicians are expected to demonstrate leadership and governance as key competencies in their various roles.^54^ The feeling of powerlessness among FPs and registrars in this study could be mitigated by FPs advocating for an improved understanding of the discipline by district managers and the provincial health departments. Family physicians should assume more active roles in engaging the university and district management about the potential impact of resource shortages on registrar training and, ultimately, on community healthcare. For example, FPs should engage facility managers at PHC level, where most of the training is conducted.^57^ This type of engagement could mitigate the lack of knowledge and understanding that could underpin inadequate support for registrar training. The district and university heads of department should support FPs in engaging district managers at various levels in raising stakeholder awareness about the necessity of adequate resource provision for effective registrar training.

### Limitations

Being an inside researcher has benefits and risks, especially when evaluating your institution.^58^ The principal author conducting the interviews may have influenced the responses of fellow FPs and the registrars she supervises in the district where she works. However, as explained, steps were taken to mitigate this. The researcher may have missed nonverbal cues in the virtual interviews, but this was unavoidable during the COVID-19 lockdown.

### Recommendations

The university advocacy and FP leadership roles could improve the provincial health department and district managers’ understanding of the value of FM training. Successful and sustainable decentralised postgraduate FM training programmes need adequate key stakeholder engagement, accelerating availability of adequate resources and skilled staff. Regular faculty support visits to support clinical trainers and standardisation of training and trainer roles across training sites is an area needing emphasis. However, faculty development through constant engagement with FP supervisors for effective trainer and supervisor roles and regular formative assessment visits to training sites is essential.

Family physicians should integrate training with service delivery instead of visualising them as separate entities. The FP leadership role assists in accessing sufficient resources for optimal training opportunities. Family physicians also need to offer peer mentoring and work as a team to enhance training and supervision creating ideal decentralised training complexes.

## Conclusion

This study aimed to explore FPs’ and registrars’ perceptions of the available resources in a decentralised postgraduate FM training programme. Improved resource provision and clinical trainer support positively influence trainer roles, impacting decentralised postgraduate FM training in district health systems. In a middle-income country like South Africa, adequate clinical trainer support helps to develop registrars into newly qualified FPs, who can better respond to the healthcare needs of communities. These findings could be of value in similar settings elsewhere.

## Appendix 1

**TABLE 1-A1:** Interview guide

Family physician	Registrar
What do you think about your skills as a clinical trainer and supervisor?	What do you think about the skills of your family physician as a clinical trainer and supervisor?
What do you think are the strengths and challenges in your role as a clinical trainer and supervisor?	What do you think are the strengths and challenges in the family physician role as a clinical trainer and supervisor?
Do you think you have adequate support from the university to improve your skills as a clinical trainer or supervisor? If you think ‘yes’ or ‘no’, please explain further	-
What do you think about the support from district management for training at your district?	What do you think about the support from district management for training at your district?
Do you think there is adequate support from the district concerning:	Do you think there is adequate support from the district concerning:
a. Equipment (if you think ‘ yes’ or ‘no’, please explain further)	a. Equipment (if you think ‘ yes’ or ‘no’, please explain further)
b. Infrastructure (if you think ‘ yes’ or ‘no’, please explain further)	b. Infrastructure (if you think ‘ yes’ or ‘no’, please explain further)
c. Wi-Fi-internet access (if you think ‘ yes’ or ‘no’, please explain further)	c. Wi-Fi-internet access (if you think ‘ yes’ or ‘no’, please explain further)
d. Funding (if you think ‘ yes’ or ‘no’, please explain further)	d. Funding (if you think ‘ yes’ or ‘no’, please explain further)
Do you think there are any other challenges on support for training in your district?	Do you think there are any other challenges on support for training in your district?
What do you think about the support from the university for the training at your district?	What do you think about the support from the university for the training at your district?
Do you think there is adequate support from the university concerning	Do you think there is adequate support from the university concerning
a. Equipment (if you think ‘ yes’ or ‘no’, please explain further)	a. Equipment (if you think ‘ yes’ or ‘no’, please explain further)
b. Infrastructure (if you think,’ yes’ or ‘no’ please explain further)	b. Infrastructure (if you think ‘ yes’ or ‘no’, please explain further)
c. Wi-Fi-internet access (if you think ‘ yes’ or ‘no’, please explain further)	c. Wi-Fi-internet access (if you think ‘ yes’ or ‘no’, please explain further)
d. Funding (if you think ‘ yes’ or ‘no’, please explain further)	d. Funding (if you think ‘ yes’ or ‘no’, please explain further)
